# Research progress on alternative kombucha substrate transformation and the resulting active components

**DOI:** 10.3389/fmicb.2023.1254014

**Published:** 2023-09-14

**Authors:** Jingqian Su, Qingqing Tan, Qian Tang, Zhiyong Tong, Minhe Yang

**Affiliations:** Fujian Key Laboratory of Innate Immune Biology, College of Life Science, Biomedical Research Center of South China, Fujian Normal University, Fuzhou, China

**Keywords:** kombucha, functional active substance, efficacy mechanism, tea beverage, probiotics

## Abstract

Kombucha is a customary tea-based beverage that is produced through the process of fermenting a mixture of tea and sugar water with symbiotic culture of bacteria and yeast (SCOBY). Traditional kombucha has various beneficial effects and can improve immunity. The significant market share of Kombucha can be attributed to the growing consumer inclination towards healthy foods within the functional beverage industry. The research focus has recently expanded from the probiotics of traditional black tea kombucha to encompass other teas, Chinese herbs, plant materials, and alternative substrates. There is a lack of comprehensive literature reviews focusing on substance transformation, functional, active substances, and efficacy mechanisms of alternative kombucha substrates. This article aimed to bridge this gap by providing an in-depth review of the biological transformation pathways of kombucha metabolites and alternative substrates. The review offers valuable insights into kombucha research, including substance metabolism and transformation, efficacy, pharmacological mechanism, and the purification of active components, offering direction and focus for further studies in this field.

## 1. Introduction

Kombucha, a traditional fermented tea beverage rich in probiotics and bio-active factors, is generally prepared from sugary tea water fermented with a symbiotic culture of bacteria and yeast (SCOBY) (Sinir et al., 2019). The composition of the symbiotic flora of kombucha is complex, and the dominant flora are composed of Acetobacter, Saccharomyces, and Lactobacillus. Previous studies on the composition of kombucha have predominantly focused on kombucha fermented with acetic acid bacteria, such as *Acetobacter xylinum*, *Gluconobacter liquefaciens*, and *Komagataeibacter intermedius*, yeast species, such as *Saccharomyces cerevisiae*, *Candida tropicalis*, and *Schizosaccharomyces pombe*, and lactic acid bacteria, for instance *Lactobacillus bulgaricus* and *Lactobacillus nagelii* (Antolak et al., 2021). Kombucha can influence various physiological functions, such as lowering blood pressure, reducing inflammation, promoting liver function, and enhancing immune resistance. Kombucha products have many metabolites, such as organic acids, polyphenols, vitamins, amino acids, protein/enzymes, and minerals (Bishop et al., 2022; Kitwetcharoen et al., 2023). The main metabolic components of kombucha are organic acids, D-saccharic acid-1,4-lactone (DSL), and tea polyphenols (Jayabalan et al., 2014). However, the probiotic effects of kombucha are independent of its unique metabolites.

Kombucha is composed of a diverse array of organic acids, including acetic acid, which can eliminate fatigue, lower blood pressure, and improve gastrointestinal health (Sinir et al., 2019). The tea polyphenols in kombucha can stabilise blood sugar, promote fat oxidation, scavenge free radicals, and delay ageing (Jayabalan et al., 2014). DSL has been found to be an effective inhibitor of β-glucosidase, an enzyme that is closely linked to the process of carcinogenesis and plays a crucial role in the prevention of cancer (Taha et al., 2020). The selection of different fermentation substrates also produces substances different from those produced using traditional kombucha. In addition to the fermentation of traditional tea leaves (black and green tea), the effects of using new fermentation substrates on the probiotic efficacy of kombucha are of growing interest (Wang et al., 2021).

Analysis of the active ingredients of kombucha and the study of the probiotic mechanisms are essential prerequisites for the industrial manufacturing and commercialization of kombucha. They are also the basis for clinical research on probiotic functional flora to prevent and treat diseases.

Herein, we reviewed recent studies on kombucha, including fermentation conditions, the composition of raw materials before and after fermentation, and the efficacy of active substances after fermentation in alternative matrices. This review will facilitate further research into the metabolism of kombucha, its efficacy mechanisms, and the development of specific kombucha-based products.

## 2. Alternative kombucha substrates

In recent years, research into alternative substrates for kombucha production has increased as most scholars have become dissatisfied with the use of tea as a substrate for traditional kombucha fermentation. The use of raw materials, such as plant leaves, fruit juices, herbs, spices, and flowers, as substitutes for tea or as co-fermentation ingredients along with black tea have been reported. These alternative substrates can produce novel fermented functional products, yield kombucha with improved organoleptic value or health properties (Table 1) (Sinir et al., 2019; Emiljanowicz and Malinowska-Pańczyk, 2020), provide carbon and nitrogen sources for the fermentation of kombucha, and alter its fermentation products to produce more beneficial and healthy bioactive beverages.

**Table 1:** Alternative raw materials for the production of black tea kombucha.

Category	Raw materials	Reference	Raw materials	References
Plant leaves	Yerba mate	Lopes et al. (2021)	*Eucalyptus camaldulensis*	Gamboa-Gómez et al. (2016)
	African mustard (*Brassica tournefortii*) leaves	Rahmani et al. (2019)	*Litsea glaucescens*	Gamboa-Gómez et al. (2016)
	Oak leaves	Vázquez-Cabral et al. (2017)	Arabica coffee leaves	Zubaidah et al. (2021a)
	Purple basil (*Ocimum basilicum* L.)	Yıkmış and Tuğgüm (2019)	Kitchen mint	Tanticharakunsiri et al. (2021)
	Soursop leaves (*Annona muricata* Linn.)	Candra et al. (2021)		
Fruit and juice	Cactus pear juice	Ayed and Hamdi (2015)	Blackthorn (*Prunus spinosa*)	Ulusoy and Tamer (2019)
	Red grape juice	Ayed et al. (2017) and Akbarirad et al. (2017)	Red raspberry (*Rubus ideaus*)	Ulusoy and Tamer (2019)
	Snake fruit (*Salacca zalacca* (Gaerth.) Voss)	Zubaidah et al. (2018)	King coconut water (*Cocos nucifera* var. aurantiaca)	Watawana et al. (2016)
	Pomegranate juice	Akbarirad et al. (2017) and Yavari et al. (2018)	Sourop (*Annona muricata*. L.)	Tan et al. (2020)
	Sour cherry juice	Akbarirad et al. (2017)	Dragon	Li et al. (2022)
	Black carrot (*Daucus carota* L. spp. *sativus* var. atrorubens Alef.)	Xiao et al. (2013)	Guava	Li et al. (2022)
	Cherry laurel (*Prunus laurocerasus*)	Ulusoy and Tamer (2019)	Indian gooseberry	Klawpiyapamornkun et al. (2023)
Herbaceous plants	Goji berry (*Lycium barbarum*)	Zhang et al. (2022) and Abuduaibifu and Tamer (2019)	*Foeniculum vulgare*	Battikh et al. (2012)
	Lemon balm (*Melissa officinalis* L.)	Četojević-Simin et al. (2012)	*Mentha piperita*	Battikh et al. (2012)
	Winter savoury (*Satureja montana*)	Vitas et al. (2020)	Wheatgrass juice	Wang et al. (2021)
	Peppermint (*Mentha* × *piperita*)	Vitas et al. (2020)	Ginger	Salafzoon et al. (2017)
	Stinging nettle (*Urtica dioica*)	Vitas et al. (2020)	Cinnamon	Shahbazi et al. (2018)
	Wild thyme (*Thymus serpyllum*)	Vitas et al. (2020)	Cardamom	Shahbazi et al. (2018)
	Elderberry (*Sambucus nigra*)	Vitas et al. (2020)	Shirazi thyme	Shahbazi et al. (2018)
	Quince (*Cydonia oblonga*)	Vitas et al. (2020)	Garlic	Pure and Pure (2016a)
	*Satureja montana* L.	Cetojevic-Simin et al. (2008)	Turmeric (*Curcuma longa*)	Zubaidah et al. (2021b)
	*Thymus vulgaris* L.	Battikh et al. (2012)	*Solanum nigrum* L. fruits	Ziska et al. (2019)
	*Lippia citriodora*	Battikh et al. (2012)	Butterfly pea flower	Permatasari et al. (2022)
	*Rosmarinus officinalis*	Battikh et al. (2012)		
Dairy products	Skim milk	Al-Dulaimi et al. (2018)	Soy whey	Tu et al. (2019)
Grain	Rice	Ahmed et al. (2020)	Corn	Francisco et al. (2021)
	Barley	Ahmed et al. (2020)		
Other	Acerola by-product	Leonarski et al. (2021)	Maise silk	Zhiwen et al. (2021)
	Citrus peel	Shin et al. (2016)	Green coffee	Aung and Eun (2021)
	Banana peel	Pure and Pure (2016b)	Butterfly pea	Shin et al. (2016)
	Common nettles	Pure and Pure (2016b)	Rose	Zhang et al. (2020)
	Laver (*Porphyra dentata*)	Aung and Eun (2021) and Aung and Eun (2022)	Jujube kernel	Zhang et al. (2020)
	Pollen	Uțoiu et al. (2018)	Dragon fruit peel	Batubara (2022)
	Pineapple peels and cores	Phung et al. (2023)		

## 3. Composition of kombucha and analogous kombucha

### 3.1. Composition of traditional kombucha

The chemical composition of kombucha is dependent on different substrate compositions, source and microbial community in the SCOBY, fermentation methods, and detection methods.

Kombucha is composed of sugars (including sucrose, glucose, and fructose), organic acids [including acetic acid, gluconic acid, glucuronic acid, lactic acid, DSL, citric acid, oxalic acid, and pyruvic acid (Jayabalan et al., 2007)], B-vitamins and vitamin C (Bauer-Petrovska and Petrushevska-Tozi, 2000), theophyllines, tea polyphenols, flavonoids, various amino acids and proteins, ethanol, biogenic amines, purine bases, hydrolytic enzymes, minerals (primarily Cu, Fe, Zn, Ni, and Mn), and metabolites secreted by yeast and bacteria (Table 2) (Malbaša et al., 2011).

**Table 2:** Biochemical components of kombucha.

Compound	Structural formula	Fermentation time (*d*)	Content	References
Sucrose	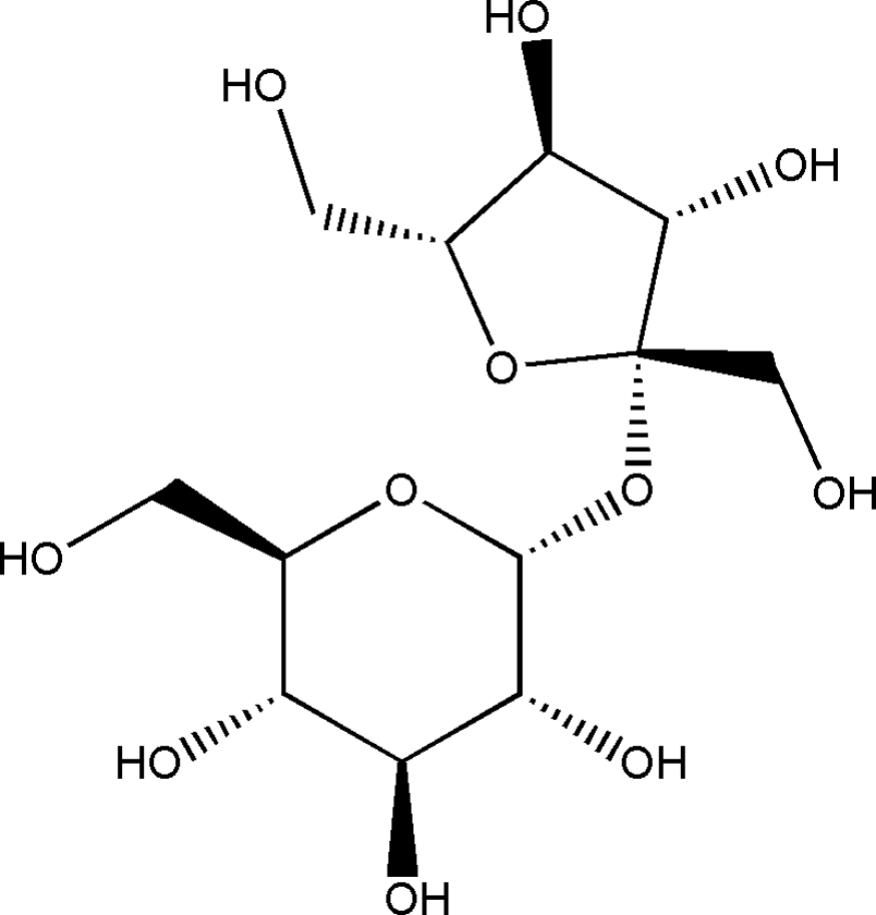	13 14	40 g/L 35 g/L	Malbaša et al. (2008)
Glucose	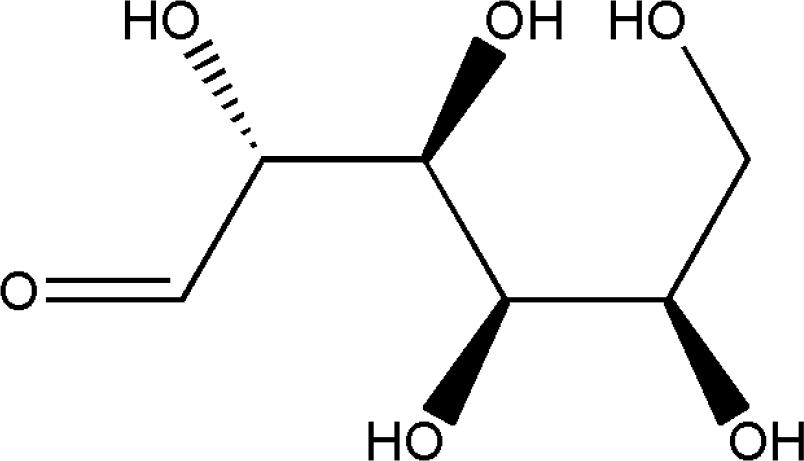	10 60	37.7 g/L 12 g/L	Chen and Liu (2000) and Neffe-Skocińska et al. (2017)
Fructose	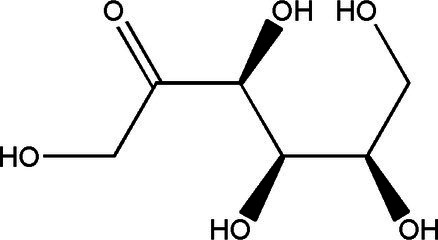	10 60	30.9 g/L 55 g/L	Chen and Liu (2000) and Neffe-Skocińska et al. (2017)
Gluconic acid	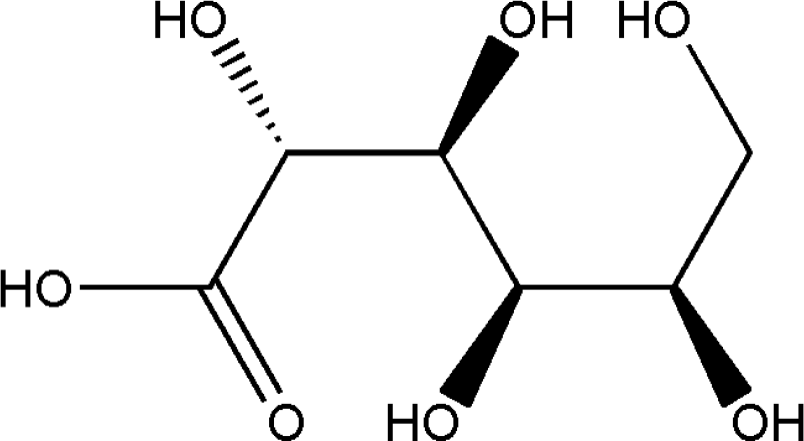	60 21	39.00 g/L 0.016 g/L	Chen and Liu (2000) and Coelho et al. (2020)
Acetic acid	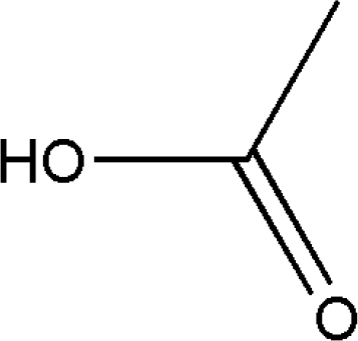	18 10 60	8.36 g/L 1.65 g/L 8.00 g/L	Jayabalan et al. (2007), Chen and Liu (2000) and Neffe-Skocińska et al. (2017)
Glucuronide	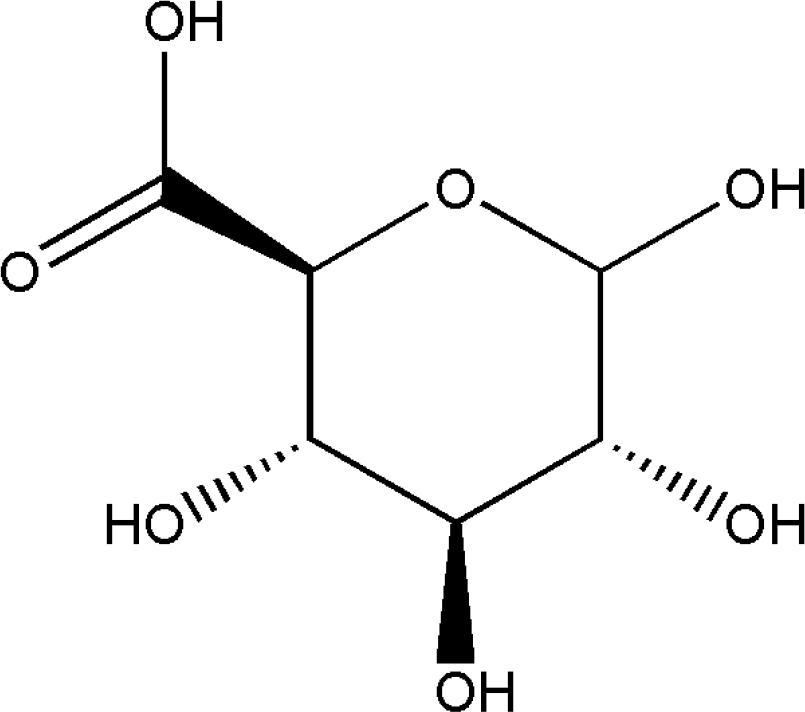	18 21 10	1.71 g/L 0.0034 g/L 0.063 g/L	Jayabalan et al. (2007), Neffe-Skocińska et al. (2017) and Petrović et al. (2000)
D-Saccharic acid-1,4-lactone	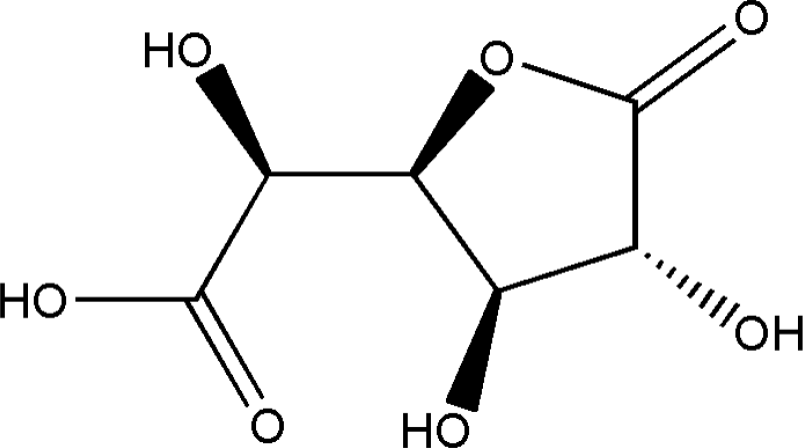	4 21	0.39 g/L 2.24 g/L	Chakravorty et al. (2016) and Chen and Liu (2000)
Citric acid	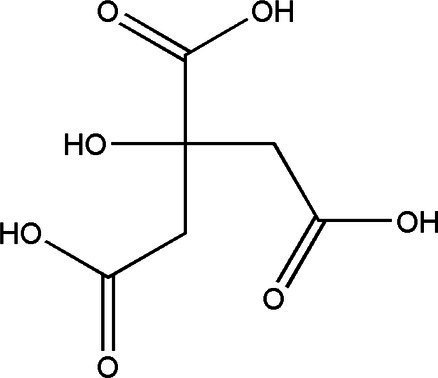	3	0.11 g/L	Jayabalan et al. (2007)
Lactic acid	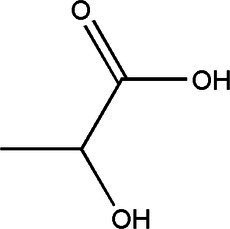	3	0.44 g/L	Jayabalan et al. (2007)
Ethanol	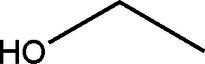	10 20	11 g/L 5.5 g/L	Chen and Liu (2000) and Neffe-Skocińska et al. (2017)
Tea polyphenols	–	14	67.2 mg/g dry weight	Gaggìa et al. (2018)

### 3.2. Material transformation of the main metabolites of traditional kombucha

Traditional kombucha fermentation utilises tea and white sugar as the main substrates. These substrates are transformed through fermentation by microorganisms, such as yeast and bacteria, which involves the gradual breakdown of sugar and a metabolic cascade that produces CO_2_ and an acidic, slightly alcoholic beverage (Chakravorty et al., 2016). In a bacteriophage fermentation environment, yeasts are the primary ethanol producers, as they produce hydrolytic enzymes (of the fructosidase class) that hydrolyse sucrose to glucose and fructose, which subsequently undergo metabolic processes leading to the production of ethanol, glycerol, and carbon dioxide. The involvement of different bacterial strains during fermentation leads to metabolic differentiation. The yeast genus *Saccharomyces* spp. can utilise glucose and produce ethanol through the glycolytic pathway, whereas the jointed yeast *Zygosaccharomyces* spp. can efficiently ferment fructose to produce ethanol. Furthermore, specific yeast strains, such as the corn wine fission yeast (*Schizosaccharomyces pombe*), have the capability to generate ethanol from malic acid or *Brettanomyces bruxellensis* in the presence of elevated levels of acetic acid in aerobic environments (Villarreal-Soto et al., 2018).

During fermentation, microorganisms interact with each other, and the ethanol produced by yeast fermentation can be used by Acetobacter as a metabolic substrate for oxidation to acetic acid. In addition to acetic acid, Acetobacter can further metabolise glucose in the fermentation broth to produce glucuronic acid, which is later metabolised to gluconic acid and converted to glucuronic acid (Villarreal-Soto et al., 2019; Martínez-Leal et al., 2020). In addition, *B. gluconii* strains have the capability to enzymatically produce L-ascorbic acid, commonly known as vitamin C, utilizing D-sorbitol as a precursor compound, which is itself derived from glucose (Mamlouk and Gullo, 2013). Depending on the specific strain, certain lactic acid bacteria have the capability to utilize glucose in either the glycolytic pathway, resulting in the production of lactic acid as the primary metabolite, or the pentose phosphate pathway, leading to the synthesis of lactic acid, ethanol, and carbon dioxide. However, when fructose is present, the production of acetic acid occurs instead of ethanol (Laureys et al., 2020).

Bacterial cellulose film is a distinctive byproduct of kombucha fermentation, which is generated by acetate bacteria through alcohol metabolism and can be removed as waste. *Komagataeibacter* spp. use glucose to synthesise bacterial cellulose, and this anabolic process involves sucrose, ethanol, and glycerol (Chawla et al., 2009; Villarreal-Soto et al., 2019). The fermentation and metabolic processes of kombucha are accomplished through mutual facilitation or constraints within each flora. The synergistic effects of the flora allow for the synthesis of certain antimicrobial metabolites, the accumulation of organic acids leading to low pH, and the production of physical barriers (cellulose membranes), and other factors contributed to the hindrance of bacterial growth among competitors (Villarreal-Soto et al., 2019).

**Figure 1:**
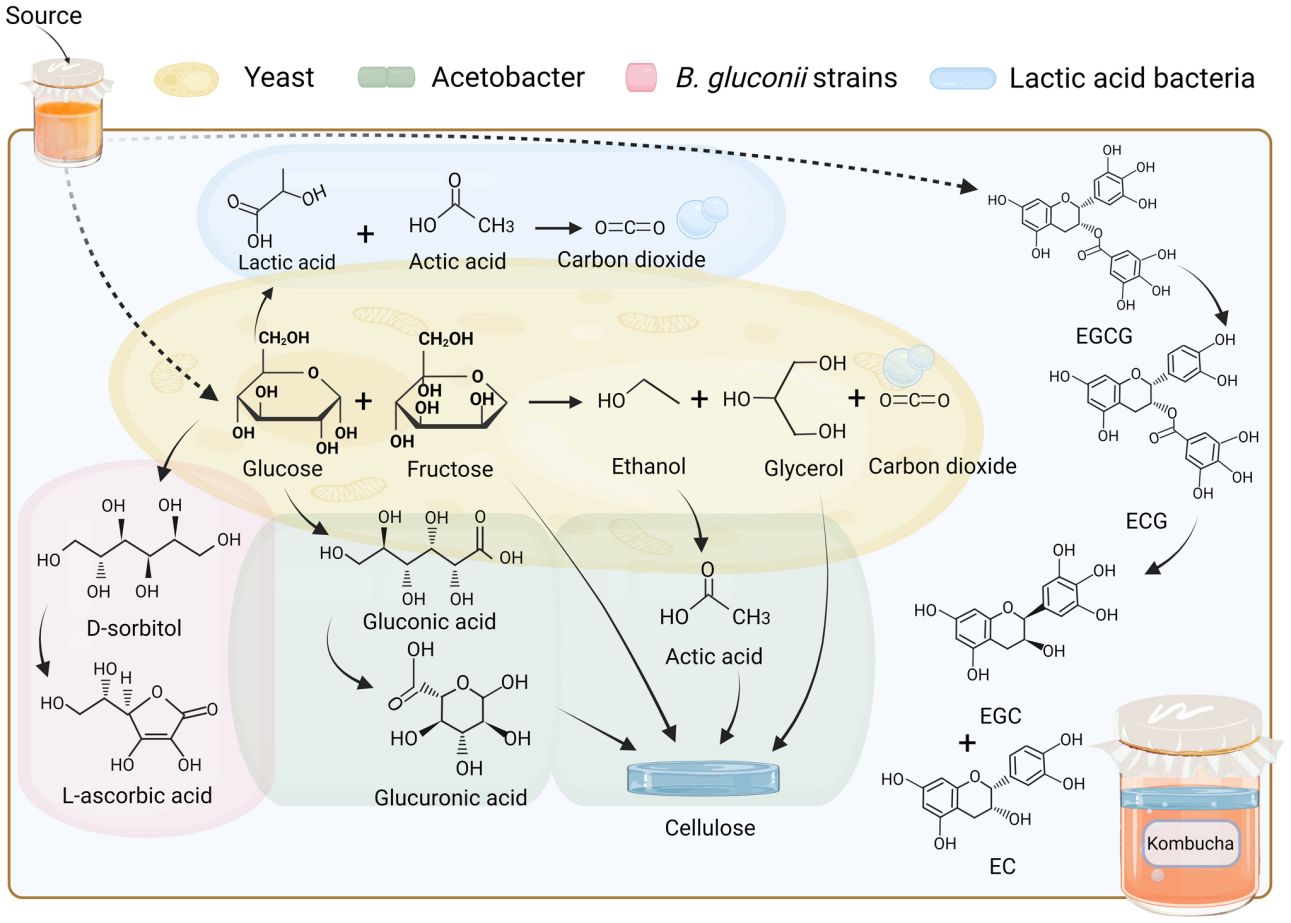
Material transformation relationship of raw materials and microbiota. Fermentation in an acidic environment or enzyme releases by bacteria and yeast may degrade or convert the complex phenolic compounds in kombucha into small biological molecules. EGCG, epigallocatechin-3-gallate; ECG, epigallocatechin gallate; EGC, epigallocatechin; EC, epicatechin.

As depicted in Figure 1, the intricate phenolic compounds present in kombucha have the potential to undergo degradation or conversion into smaller biological molecules through the process of fermentation occurring within an acidic milieu, or through the enzymatic activities released by bacteria and yeast. For example, the observed elevation in overall catechin content in green and black tea kombucha subsequent to fermentation can be ascribed to the biotransformation process wherein epigallocatechin-3-gallate (EGCG) undergoes conversion into epigallocatechin gallate (ECG) and epicatechin (EC), by enzymes released by microbial communities in an acidic environment. EGCG is hydrolysed into smaller molecules and converted into epigallocatechin (EGC), EGCG, and EC (Jayabalan et al., 2008). Theaflavins and thearubigin are complex polyphenol derivatives found in black tea and are associated with colour changes in tea (Martínez-Leal et al., 2018). The lighter colour of a fully fermented kombucha may be attributable to the conversion of theaflavins to theobromine (Jayabalan et al., 2007).

### 3.3. Substance transformation of alternative raw materials after fermentation by kombucha

Traditional kombucha is prepared from black tea and white sugar, which are the primary raw materials used in microbial fermentation. However, in the last three decades, considerable research has resulted in the replacement of black tea and white sugar with other teas, herbs, plant materials, and sugars. The pharmacological products of fermentation depend on the active substances and their amount in the broth. The metabolites generated during kombucha fermentation of alternative substrates exhibit a strong correlation with the characteristics of the initial raw materials (Table 3).

**Table 3:** Transformation of the black tea alternative substances after fermentation.

Raw materials	Fermentation time (*d*)	Fermentation temperature (°C)	Unfermented	Fermented	References
Green tea	0, 1, 3, 6,7, 9, 12, 14, 15	25, 28 ± 1	Polyphenolic catechins [epigallocatechin gallate (EGCG), epigallocatechin (EGC), epicatechin gallate (ECG), and epicatechin (EC)], polyphenols, flavonoids	Theaflavins, glucuronic acid, gluconic acid, DSL, acetic acid↑, ascorbic acid, total phenols↑, total flavonoids↓, alcohol↑,	Kaewkod et al. (2019); Jakubczyk et al. (2020)
Oolong tea	0, 3, 6, 9, 12, 15	25	Catechins	Glucuronic acid, gluconic acid, DSL, acetic acid, ascorbic acid,total phenols	Kaewkod et al. (2019)
Red tea	1, 7, 14	28 ± 1	Polyphenols, flavonoids	Total flavonoids↓, total phenols, alcohol↑,acetic acid↑	Jakubczyk et al. (2020)
White tea	1, 7, 14	28 ± 1	Polyphenols, flavonoids	Total flavonoids↓, total phenols↑, alcohol↑	Jakubczyk et al. (2020)
Yerba mate	7, 14, 21, 35	25	Polyphenolic acids [chlorogenic acid, caffeic acid, 3, 4-dicafeoylquinic acid, 3, 5-dicafeoylquinic acid, xanthines (caffeine and theobromine)], flavonoids (quercetin, kaempferol, and rutin), amino acids, minerals (P, Fe, and Ca), vitamins (C, B1, and B2), saponins, alkaloids	Caffeic acid, 3-caffeoylquinic acid, 4-caffeoylquinic acid, 5-caffeoylquinic acid, 3,4-dicaffeoylquinic acid, 3,5-dicaffeoylquinic acid, 4,5-dicaffeoylquinic acid, phenolic acids, theobromine, caffeine, rutin	Ziemlewska et al. (2021)
Cactus pear juice	15	30	Vitamins, amino acids, minerals, polyphenols, betalains, indicaxanthin, flavonoids	Total phenols↑, betalains (betacyanin↑)	Ayed and Hamdi (2015)
Red grape juice	15	30	Vitamins, minerals, carbohydrates, edible fibre, polyphenols [phenolic acids, resveratrol, proanthocyanidins, flavonoids(anthocyanin)]	Organic acids, total phenols, anthocyanins	Ayed et al. (2017) and Akbarirad et al. (2017)
Snake fruit	14	25	Vitamins, minerals, dietary fibre	Total phenols, tannic acid, total flavonoids, organic acids	Zubaidah et al. (2018)
Pomegranate juice	14	37	Phenol-carboxylic acids, anthoxanthins (flavonoids, anthocyanins), astringent-polyphenolic compounds (tannins), antioxidants	Total acidity, glucuronic acid, reducing sugars	Yavari et al. (2018)
Cherry laurel juice	40 h	28 ± 2	Vitamin C, Phenolic substances (anthocyanins)	Total phenols, total monomeric anthocyanins, total acidity	Ulusoy and Tamer (2019)
Blackthorn juice			Polyphenolic compounds, vitamin C		
Red raspberry juice			Polyphenols, anthocyanins, ellagitannins		
Black carrot juice			Phenolic compounds, vitamins C and E		
King coconut water	7	24 ± 3	Sugars, vitamins (vitamin B complex, vitamin C), amino acids and minerals, carbohydrate	Ethanol, total acid, total phenolic (ferulic acid ↑, *p*-coumaric acid ↑)	Watawana et al. (2016)
Goji berry	1–14	28 ± 2	Red goji berry: carotenoids (beta-carotene, lutein, lycopene, zeaxanthin, zeaxanthin dipalmitate), polysaccharides, vitamins (ascorbic acid tocopherol), minerals, fatty acids, betaine, peptidoglycans Black goji berry: purple anthocyanins, proteins, free amino acids, essential oils, organic acids, carbohydrates, vitamin C, B1, B2, minerals, alkaloids	Total acidity ↑, total phenolics ↑	Abuduaibifu and Tamer (2019)
Yarrow	7	25	Achilleine, apigenin, luteolin, azulene, camphor, coumarin, inulin, menthol, quercetin, rutin, succinic, salicylic, caffeic acids, thujone	Organic acids (oxalic acid ↑, formic acid ↑, acetic acid ↑, succinic acid ↑, malic acid ↑, citric acid ↑), total phenols ↑, flavonoids ↓, vitamin C ↑	Vitas et al. (2018)
Wheatgrass juice	12	29 ± 1	Chlorophyll, vitamins (A, C, E), bioflavonoids, minerals (iron, calcium, magnesium), phenolics (ferulic acid, vanillic acid)	Total phenolic, total flavonoids, total anthocyanin	Wang et al. (2021)
Ginger	10	25	Gingerols, shogaols, zingerone, paradols	–	Salafzoon et al. (2017)
Cinnamon	1–16	28	Cinnamaldehyde, eugenol, coumarin	Organic acid ↑, Total phenolic ↑, total flavonoid ↑	Shahbazi et al. (2018)
Cardamom			–		
Shirazi thyme			Carvacrol		
Garlic	21	25	Organosulfur compounds	Total phenolic ↑	Pure and Pure (2016a)
African mustard leaves	14	25–30	Glucosinolates, polyphenols, carotenoïds, vitamins	Ethyl acetate ↑, sugar ↓, ethanol and acetic acid (↑ then ↓), total phenols ↑	Rahmani et al. (2019)
Oak leaves	7	25	Polyphenols (catechin, quercetin, kaempferol, naringin, naringenin, ellagic acid), tannins (vescalagin, castalagin), proanthocyanidins	Polyphenols, organic acids, sugars, gluconic acid, glucuronic acid	Vázquez-Cabral et al. (2017)
Purple basil	10	24 ± 3	Polyphenols, aromatic compounds	Polyphenols, flavonoids	Yıkmış and Tuğgüm (2019)
Soy whey	7	28	Protein, oligosaccharide, isoflavones, organic acid, minerals	Total reducing sugars, total flavonoids ↑, glucuronic acid ↑, organic acids ↑, isoflavones ↑, volatile components	Tu et al. (2019)
Rice	12	28 ± 2		Total acidity, ethanol ↑, total protein ↑, total phenol	Ahmed et al. (2020)
Barley					
Acerola by-product	0–15	30	Vitamin C, polyphenols	Total phenolic ↑, ethanol ↑, acetic acid ↑, cellulose ↑, vitamin C ↑	Leonarski et al. (2021)
Banana peel	21	25	-	Total phenolic contents ↑, protein ↑	Pure and Pure (2016b)
Common nettles					

↑ indicates an increase in content and ↓ indicates a decrease in content.

### 3.4. Antibacterial property

Kombucha beverages produced through the fermentation of alternative raw material extracts are known to have antibacterial potential. The antibacterial profile of various substrates after fermentation with kombucha (Table 4) is influenced by different factors, including fermentation time, raw materials, temperature, and kombucha strains. The fermentation of kombucha is often considered a key factor contributing to its antibacterial activity. The competitive growth advantage of the dominant flora of traditional kombucha and the production of secondary metabolites, such as tea polyphenols, organic acids, and ethanol, can impede the growth and proliferation of pathogenic bacteria and fungi. Ayed et al. found that the growth of certain microbes, such as *Staphylococcus aureus*, *Bacillus cereus*, and *Staphylococcus epidermidis*, was found to be inhibited by fermented cactus pear juice; whereas unfermented pear fruit cactus juice had no antibacterial effect, and this inhibitory property was lost when the samples were neutralized (Ayed and Hamdi, 2015). The organic acids, specifically acetic acid, were identified as the primary contributors to the observed antimicrobial activity (Ayed and Hamdi, 2015). Četojević-Simin et al. employed an agar diffusion method to investigate the antimicrobial properties of lemon balm tea. The results indicated that unfermented lemon balm tea, with a dry weight concentration of 5 g/L, did not demonstrate any antimicrobial activity. However, the fermented lemon balm tea kombucha exhibited significant antimicrobial activity against prokaryotic microorganisms (G+ bacteria and G− bacteria) (Četojević-Simin et al., 2012). While no inhibitory activity was observed against fungi, yeast, or mould, the observed inhibition had similar activity to an acetic acid solution, which inferred that acetic acid served as the primary inhibitory agent within the kombucha beverage. The presence of other heat-resistant antibacterial components was verified by neutralizing with heating (Četojević-Simin et al., 2012). Vitas et al. (2018) identified significant antimicrobial properties in subcritical aqueous extracts of yarrow after fermentation; the potential antimicrobial substances were likely organic acids, phenolic compounds of plant origin, enzymes, proteins, and bacteriocins produced through fermentation.

**Table 4:** Antibacterial effects of alternative raw materials after fermentation.

Raw materials	Antibacterial spectrum	Detection method	Antibacterial ingredients	References
Cactus pear juice	*Staphylococcus aureus*, *Bacillus cereus*, *Staphylococcus epidermidis*, *Enterococcus faecalis*, *Escherichia coli*, *Klebsiella pneumoniae, Pseudomonas aeruginosa*	Agar diffusion method	Acetic acid	Ayed and Hamdi (2015)
Red grape juice	*Escherichia coli*, *Pseudomonas aeruginosa*, *Klebsiella pneumoniae*, *Staphylococcus aureus*, *Enterococcus faecalis*, *Bacillus cereus, Staphylococcus epidermidis*	Agar diffusion method	Acetic acid, other metabolites	Ayed et al. (2017)
Snake fruit	*Staphylococcus aureus*, *Escherichia coli*	Agar diffusion method	Acetic acid, natural bioactive compounds of the snake fruit, phenolic compounds, flavonoids	Zubaidah et al. (2018)
Lemon balm	*Salmonella enteritidis*, *Escherichia coli*, *Proteus mirabilis*, *Pseudomonas aeruginosa*, *Erwinia carotovora*, *Staphylococcus aureus*, *Bacillus cereus*	Agar diffusion method	Acetic acid, thermostable antimicrobial components	Četojević-Simin et al. (2012)
Yarrow	*Staphylococcus aureus Staphylococcus aureus, Klebsiella pneumoniae, Escherichia coli-* *Bacillus ichia coli, Proteus vulgaris, Proteus mirabilis, Bacillus subtilis, Candida albicans, Aspergillus niger*	Minimum inhibitory concentration	Organic acids, plant-derived phenolic compounds, enzymes, proteins, bacteriocins	Vitas et al. (2018)
Cinnamon	*S. aureus*, *B. cereus*, *E. coli*, *S. typhimurium*	Minimum inhibitory concentration	Organic acid (mainly acetic acid), flavonoid	Chawla et al. (2009)
Cardamom				
Shirazi thyme				
Garlic	*S. saprophyticus*, *S. aureus*, *S. epidermidi*, *B. stearothermophilus*, *S. typhimurium, E. coli, P. aeroginosa*	Paper diffusion method	Active chemicals of garlic	Pure and Pure (2016a)
Soy whey	*Staphylococcus aureus*, *Bacillus subtilis*, *Escherichia coli*	Agar diffusion method	Organic acid (acetic acid), large proteins, polyphenols (flavonoids), bacteriocins, enzymes	Tu et al. (2019)
Turmeric	*Escherichia coli*	Paper diffusion method	Curcuminoids, terpene derivatives (sesquiterpenes and monoterpenes)	Zubaidah et al. (2021b)
*Lycium barbarum*	*Escherichia coli*, *Staphylococcus aureus*	Agar well diffusion method	Organic acids, *L. barbarum* polysaccharides (LBP), metabolites	Zhang et al. (2022)

The primary antimicrobial agent found in kombucha is acetic acid. The phenomenon encompasses the capacity of the undissociated acid to undergo unhindered diffusion across the lipid bilayer and subsequently release protons from the cytoplasm, thereby reducing the pH of the cytoplasm. This process also involves the integration of the undissociated acid within the lipid bilayer under conditions of low external pH, ultimately resulting in the accumulation of anions. The antimicrobial activity of kombucha is primarily attributed to two mechanisms: cytoplasmic acidification and the accumulation of free acid anions at toxic concentrations (Jayabalan et al., 2008). The acidification of the bacterial cytoplasm can impede bacterial growth through the inhibition of glycolysis, hindrance of active transport, or disruption of signal transduction (Roe et al., 2002).

In addition to organic acids, other components introduced by alternative raw materials cannot be ignored in bacterial inhibition. Some phenolic compounds with antimicrobial activity can affect the hyperacidification of the plasma membrane interface.

The integrity of this membrane can be compromised either by the H+-ATPase enzyme necessary for ATP production or through interaction with bacterial DNA, resulting in modifications to bacterial physiology and impeding cellular proliferation. The presence of *Lycium barbarum* polysaccharides in the fermentation broth exerted a pronounced inhibitory impact on both gram-positive and gram-negative bacteria. This effect was attributed to the rapid disruption of cellular membranes, thereby impeding the passage of essential nutrients and metabolites across the bacterial cells, ultimately leading to their inhibition (Zhang et al., 2022). Zubaidah et al. (2021c) revealed that the antibacterial activity of turmeric kombucha increased with the turmeric concentration and decreased after reaching a certain level. The antibacterial components of turmeric kombucha differ from turmeric concentrations; the curcuminoid compounds in turmeric and its essential oil act as antimicrobial agents by inhibiting the metabolism of microorganisms. In addition, terpene derivatives (sesquiterpenes and monoterpenes) in essential oils can disrupt the structure of bacterial cell membranes (Zubaidah et al., 2021b). Shahbazi et al. (2018) observed a notable reduction in the minimal inhibitory concentration (MIC) of cinnamon fermentation broth with decreasing pH; cinnamon components also disrupted the cytoplasmic membranes of gram-positive and gram-negative bacteria and reduced intracellular ATP concentrations. Pure and Pure (2016b) reported that gram-positive bacteria were more sensitive to garlic extracts than gram-negative bacteria. This phenomenon can be attributed to the influence of the lipid constituents present in the cell wall, which hinder the penetration of garlic’s active compounds into the cells.

In summary, fermented broth often combines the antibacterial properties of the raw material and the microbial strain, with organic acids being the active components. Fermentation creates an acidic environment for drugs and influences the antibacterial properties of raw materials, and the active substances of the raw materials also contribute to the antibacterial power. However, owing to the differences in strains, the intricate composition of strains, and diverse culture environments, it is difficult to evaluate the chemical composition and core flora associated with the inhibitory effects reported in each study, making it difficult to achieve consistent inhibitory activity across different studies. Pure and Pure (2016b) tested various samples using the disk diffusion method against *S. typhimurium, S. aureus, E. coli, S. saprophytic, B. saprophyticus*, and *Pseudomonas aeruginosa*. The authors found that the infused and fermented samples of common nettles, banana peel, and black tea did not demonstrate any antibacterial efficacy against the tested bacteria; this was thought to be attributed to the differences in the concentration of the aqueous extracts and the assay method (Pure and Pure, 2016b). Therefore, the development of kombucha fermentation broth as a natural biological preservative requires further work. More in-depth research on the antibacterial mechanisms of kombucha is required to optimise the strain composition, regulate the fermentation process, and actively develop kombucha products that are suitable for consumption and have clear health benefits.

### 3.5. Antioxidant effects

The fermentation process can enhance the antioxidant properties of Kombucha, thereby rendering it an excellent source of antioxidants (Table 5). Gamboa-Gómez et al. (2016) evaluated the effect of kombucha fermentation on the antioxidant activities of *Litsea glaucescens* and *Eucalyptus camaldulensis,* using three antioxidant activity indicators, namely thiobarbituric acid reactive substances (TBARS), α, α-diphenyl-ß-picrylhydrazyl (DPPH), and nitric oxide (NO). The researchers discovered that the process of kombucha fermentation enhanced the capacity of natural herbal infusions to effectively scavenge free radicals and inhibit lipid peroxidation (Gamboa-Gómez et al., 2016). Sun et al. used traditional kombucha as a control to test against a 1:1 mixture of wheatgrass juice and brown sugar tea and found that the modified fermentation broth exhibited higher levels of phenolic acid content and oxidative radical absorption capacity compared to the traditional kombucha. Notably, the DPPH scavenging rate reached 90% (Sun et al., 2015).

**Table 5:** Antioxidant effects of alternative raw materials after fermentation.

Raw material	Bioactive compound	Experimental method	References
Yerba mate	Polyphenols, alkaloids, flavonoids	DPPH, ABTS, ROS/fibroblasts, and keratinocytes	Chen and Liu (2000)
Cactus pear juice	Betalains, polyphenols	DPPH, ABTS	Ayed and Hamdi (2015)
Red grape juice	Total phenols, anthocyanins	DPPH, ABTS	Ayed et al. (2017)
Snake fruit	Phenolics, tannins and flavonoids	DPPH	Shahbazi et al. (2018)
Cherry laurel juice	Total phenolics	DPPH, FRAP, CUPRAC	Ulusoy and Tamer (2019)
Blackthorn juice	Total phenolics		
Red raspberry juice	Total phenolics, anthocyanins, ellagitannins		
Black carrot juice	Total phenolics		
King coconut water	Phenolic compounds (ferulic acid, *p*-coumaric acid), Vitamins (vitamin B complex, vitamin C)	DPPH, ABTS, FRAP, ORAC	Watawana et al. (2016)
Goji berry	Total phenolic	DPPH, FRAP, CUPRAC	Abuduaibifu and Tamer (2019)
Winter savoury, peppermint, stinging nettle, wild thyme, elderberry, quince	Total phenols, total flavonoids, catalase	Catalase activity, reducing power, DPPH, hydroxyl radical scavenging ability	Vitas et al. (2020)
Yarrow	Phenols, organic acids, vitamin C	DPPH, reducing power	Vitas et al. (2018)
Wheatgrass juice	Phenols, flavonoids	DPPH, ABTS, ORAC	Wang et al. (2021)
Ginger	Emiljanowicz and Malinowska-Pańczyk (2020)-gingerol, Emiljanowicz and Malinowska-Pańczyk (2020)-shogaol	SOD, catalase activity/breast cancer	Salafzoon et al. (2017)
Cinnamon	Cinnamic acid, eugenol, coumarin Phenols	DPPH	Shahbazi et al. (2018)
Cardamom			
Shirazi thyme			
Garlic	Phenols, gluconic acid, glucuronic acid, vitamins, amino acids	DPPH	Pure and Pure (2016a)
African mustard leaves	Total phenolic, vitamin C, vitamin A	DPPH	Rahmani et al. (2019)
Oak leaves	Polyphenols	THP-1 cells	Vázquez-Cabral et al. (2017)
Purple basil	Phenolic compounds, flavonoids	DPPH, CUPRAC	Yıkmış and Tuğgüm (2019)
Soy whey	Isoflavone aglycones, iron chelated compounds, Polyphenols, gluconic acid, glucuronic acid	DPPH, ABTS, FRAP, reducing power	Tu et al. (2019)
Rice	Total phenolic compounds	DPPH	Ahmed et al. (2020)
Barley			
Acerola by-product	Polyphenols, vitamin C	DPPH	Leonarski et al. (2021)
Banana peel	Acetic acid, polyphenols	DPPH	Pure and Pure (2016b)
Common nettles			
Pollen	Polyphenols, flavonoids	DPPH, TEAC	Uțoiu et al. (2018)
Green coffee	Phenols, flavonoids	DPPH, ROS/keratinocyte (HaCaT), and fibroblast (BJ) cells, SOD	Zofia et al. (2020)
*Lycium barbarum*	Phenols, flavonoids, polysaccharides	DPPH, reducing power, SOD	Zhang et al. (2022)
Butterfly pea	Flavonoids, tannins, saponins, phenols, organic acids, DSL	DPPH	Christiani Dwiputri and Lauda Feroniasanti (2019)
Rose, jujube kernel	Polyphenols, flavonoids, quercetin, gallic acid	DPPH, reducing power, SOD	Zhang et al. (2020)
Butterfly pea flower	Polyphenolic compounds (kaempferol, rutin, quercetin)	ABTS	Permatasari et al. (2022)

ABTS, 2,2′-azino-bis (3-ethylbenzothiazoline-6-sulfonate); CUPRAC, cupric reducing antioxidant capacity; DSL, D-saccharic acid-1,4-lactone; FRAP, ferric ion reducing antioxidant power; ORAC, oxygen radical absorbance capacity; ROS, reactive oxygen species; SOD, superoxide dismutase; TEAC, tetraethylammonium chloride.

Polyphenols are the main antioxidants in the kombucha fermentation broth, and these entities possess the ability to readily donate hydroxyl hydrogen due to their resonance stabilization (Miller et al., 1993). This hydrogen supply enhances the DPPH-scavenging ability of the phenolic compounds. The increase in antioxidant properties of alternative raw materials for fermentation is usually associated with increased total phenol content, which is attributable to enzymatic synthesis and acid hydrolysis (Ayed et al., 2017). During the process of fermentation, microorganisms, specifically *Acetobacter* and *Saccharomyces*, present in kombucha, secrete enzymes that break down polyphenols into smaller compounds possessing potent antioxidant properties. Enzymes secreted by the kombucha SCOBY cleave flavonoid ring structures and/or phenolic conjugation sites, leading to structural rearrangements. Common rearrangements include the depolymerisation of theaflavins and theobromine and the cleavage of gallic acid into gallic acid (Chen and Liu, 2000). The potential outcome of these rearrangements is an enhancement in the bioavailability of phenolic compounds, specifically gallic acid, EC, and ECG 32.

The antioxidant properties of alternative raw materials stem from the differences in their total phenol contents and the specific substrates that generate antioxidant components during fermentation. Ayed et al. (2017) used red grape juice as an alternative substrate, and the fermentation process generated beneficial compounds that enhanced the antioxidant properties of the beverage. Ulusoy and Tamer (2019) discovered that the predominant contributors to the antioxidant activity of raspberries were anthocyanins and ellagitannins, accounting for 25% and 52% of the overall antioxidant activity, respectively. In addition, Zhang et al. (2022) found that the kombucha SCOBY has the potential to alter the composition of polysaccharides and the ratio of monosaccharides to polysaccharides after fermentation with *Lycium barbarum*, which affected its antioxidant activity.

### 3.6. Anti-inflammatory effects

**Figure 2:**
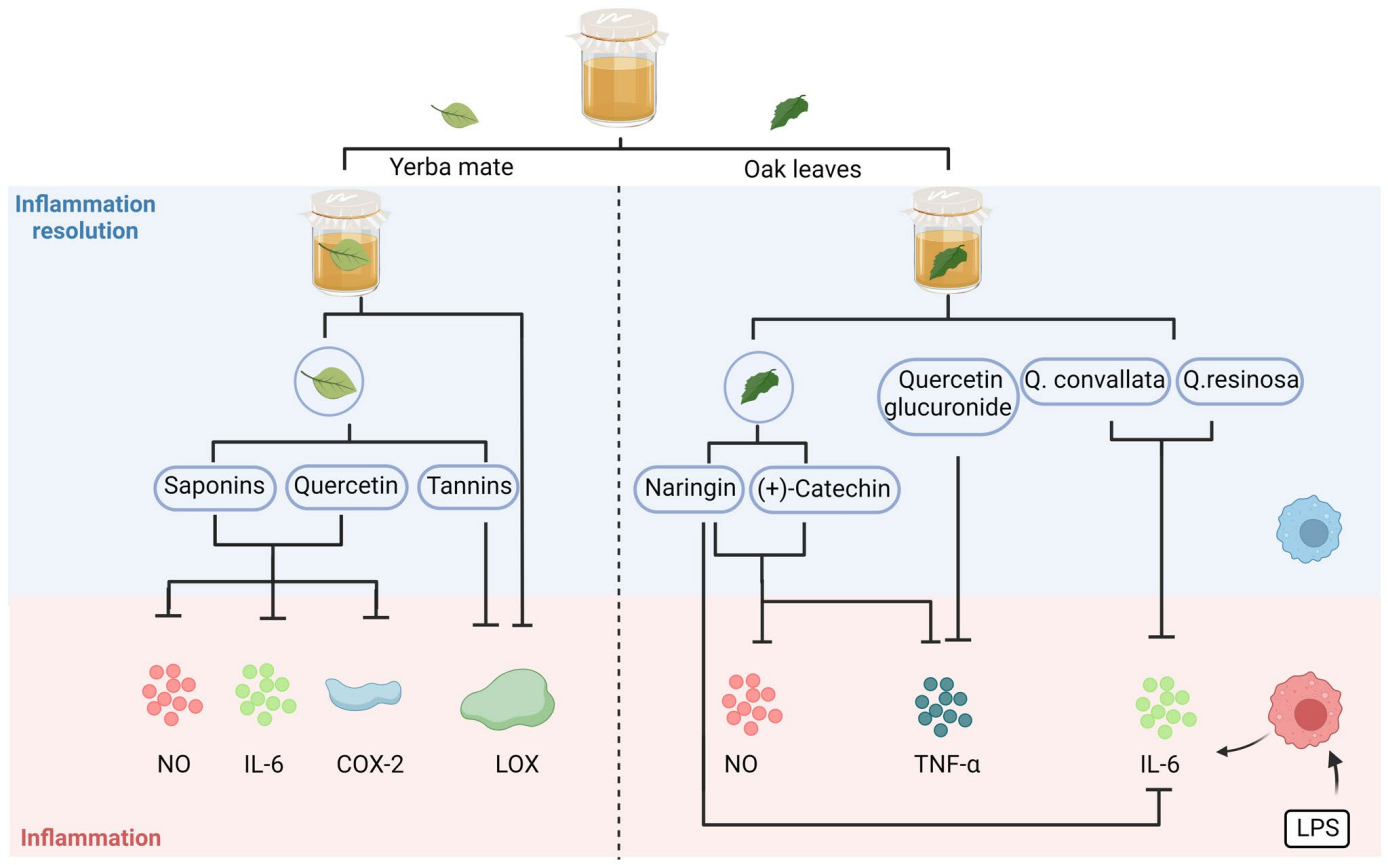
Anti-inflammatory activities of alternative substrates after fermentation. The anti-inflammatory properties of kombucha are attributed to a combination of beneficial compounds generated by the flora’s biotransformation and metabolism. NO, nitric oxide; IL-6, interleukin-6; COX-2, cyclooxygenase-2; LOX, lipoxygenase; TNF-α, tumour necrosis factor alpha; LPS, lipopolysaccharides.

As depicted in Figure 2, the anti-inflammatory effect of kombucha can be attributed to the beneficial components, including flavonoids and phenolic acids (gallic acid, catechins, and theaflavins), produced by the biotransformation and metabolism of the flora. The ameliorating effect of kombucha (15 mg/kg) on gastric ulcers was comparable to that of the positive control drug, omeprazole (3 mg/kg). Theaflavin was the main anti-inflammatory component, and the healing rate of theaflavin (1 mg/kg) in mice over 7 days was 81.4% (Banerjee et al., 2010). Many kombucha fermentation broths prepared using alternative raw materials exhibit anti-inflammatory properties. Kanno et al. (2006) used tea made from oak leaves as a substrate for preparing kombucha fermentation broth, and the broth effectively downregulated NO production and exhibited a substantial decrease in the expression of tumour necrosis factor-alpha (TNF-α) and interleukin-6 (IL-6) in lipopolysaccharide (LPS)-stimulated macrophages (THP-1). The presence of naringin in oak demonstrated a significant inhibition of LPS-induced nitric oxide (NO) production, as well as the expression of inflammatory gene products, including inducible nitric oxide synthase (iNOS), TNF-α, and IL-6 (Kanno et al., 2006); moreover, (+)-catechin present in oak inhibited LPS-stimulated NO and TNF-α production in macrophages (Guruvayoorappan and Kuttan, 2008). The phenolic content of Kombucha, which is produced through the fermentation of oak leaf tea, contributes to its notable anti-inflammatory and antioxidant properties (Vázquez-Cabral et al., 2017). Ziemlewska et al. (2021) found that yerba mate extracts used as an alternative raw fermentation material showed potent inhibition of lipoxygenase (LOX) after 14 and 21 days of fermentation. The enzyme LOX plays a crucial role in the synthesis of leukotrienes, which are known as mediators of the pro-inflammatory response. Therefore, the regulation of LOX activity holds significant importance in the treatment of inflammation (Nworu and Akah, 2015; Oguntibeju, 2018).

Quercetin and saponins from yerba mate extract are the main components responsible for their anti-inflammatory effects, and these substances can reduce the production of IL-6, cyclooxygenase-2 (COX-2), and NO—the main mediators of the inflammatory process. Additionally, the tannins in yerba mate extracts have an inhibitory effect on LOX activity (Ziemlewska et al., 2021).

### 3.7. Anti-diabetic effects

**Figure 3:**
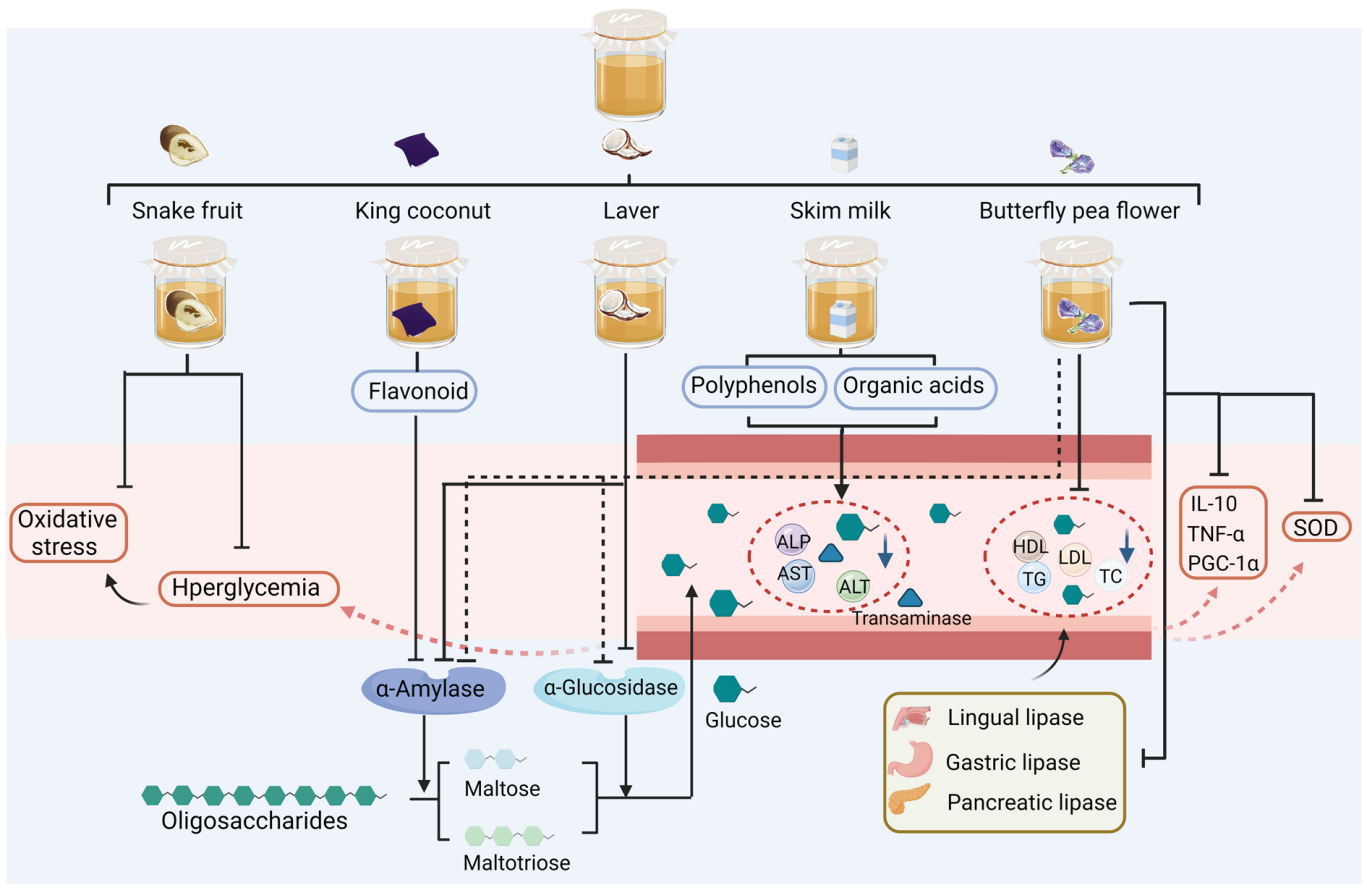
Sugar-reducing activity of alternative substrates after fermentation. ALP, alkaline phosphatase; ALT, alanine aminotransferase; AST, aspartate transaminase; HDL, high-density lipoprotein cholesterol; LDL, low-density lipoprotein cholesterol; TC, total cholesterol, TG, triglyceride; PGC-1α, peroxisome proliferator-activated receptor-γ coactivator 1-α; TNF-α, tumour necrosis factor alpha; IL-10, interleukin-10.

Diabetes mellitus is a metabolic disorder characterized by inadequate insulin secretion or islet insufficiency. As shown in Figure 3, studies on the hypoglycaemic effects of kombucha have mainly focused on the analysis and histological observations of the biological activities of key enzymes in the glucose metabolic pathway; only a few in-depth studies have been reported on the associated molecular mechanisms. Zubaidah et al. (2019) demonstrated that the fermentation process of snake fruit extracts into kombucha effectively mitigated oxidative stress and provided stability to fluctuations in fasting blood glucose levels within a streptozotocin-induced diabetic model. This glucose-lowering effect was comparable to that of metformin hydrochloride—the “king” glucose-lowering drug (Zubaidah et al., 2019). Watawana et al. (2016) found that king coconut water fermented into kombucha resulted in the enhanced inhibition of starch hydrolases. The inhibitory activity of both α-amylase and glucosidase was enhanced by the fermentation process, with a greater enhancement observed in α-amylase inhibitory activity compared to α-glucosidase inhibitory activity. The enzyme α-amylase exerts its action prior to α-glucosidase, thus hindering the reaction rate of α-glucosidase and impeding the release of glucose into the physiological system. This outcome proves advantageous as it demonstrates a beneficial anti-hyperglycemic effect (Watawana et al., 2016). Aung and Eun (2021) showed that the fermentation broth of laver, as an alternative raw material, inhibited amylase activity *in vitro*. Fermented laver kombucha, which contained important flavonoid compounds, enhanced α-amylase inhibitory activity at 25°C (Aung and Eun, 2021). Al-Dulaimi et al. (2018) explored the effects of kombucha fermentation with skim milk as a substrate on serum glucose concentrations, total lipid profiles, and body weight in male rats. Skim milk fermentation reduced blood glucose, total lipid, alanine aminotransferase (ALT), aspartate transaminase (AST), and alkaline phosphatase (ALP) in the rats and exerted beneficial effects on the human liver and overall health. This was attributable to the combined effects of polyphenols (such as flavonoids and catechins), vitamin E, and organic acids in the fermentation broth (Al-Dulaimi et al., 2018). Permatasari et al. (2022) found that butterfly pea flower kombucha (KBPF) inhibited α-amylase and α-glucosidase activities, achieving similar levels of inhibition to the acarbose control at concentrations of 50–250 μg/mL. *In vivo*, KBPF administration (130 mg/kg BW) significantly alleviated the metabolic disturbances induced by a high-fat diet through the modulation of glucose level, oxidative stress markers (superoxide dismutase), metabolic enzymes (lipases and amylases), inflammatory markers (PGC-1α, TNF-α, and IL-10), and lipids, such as total cholesterol (TC), triglyceride (TG), low-density lipoprotein cholesterol (LDL), and high-density lipoprotein cholesterol (HDL). In addition, KBPF had a positive effect on the phyla Bacteroidetes and Firmicutes (Permatasari et al., 2022).

### 3.8. Skincare applications

**Figure 4:**
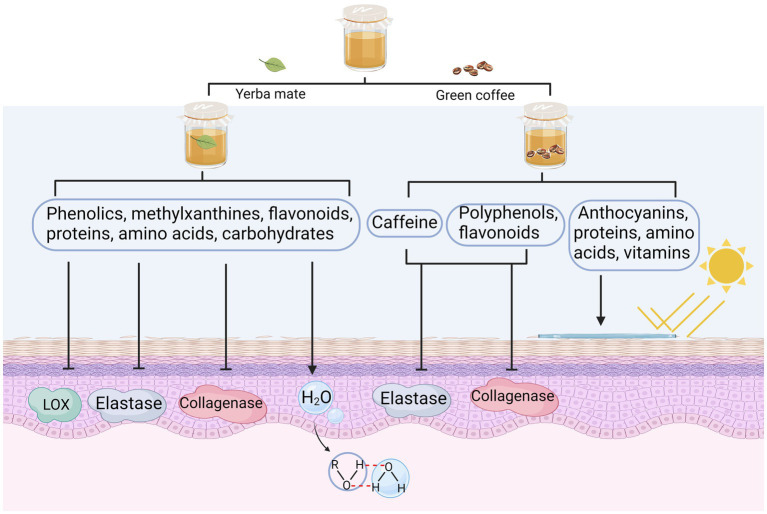
Skincare activity of alternative substrates after fermentation. LOX, lipoxygenase.

As shown in Figure 4, Ziemlewska et al. (2021) determined the skin care effects of fermented yerba mate kombucha on keratinocytes and fibroblast cell lines. They found that fermentation strongly inhibited lipoxygenase, collagenase, and elastase activities. Furthermore, the researchers noted a sustained moisturizing effect subsequent to the topical administration. The fermentation solution comprised of phenolic acids, methylxanthines, and flavonoids, which actively contributed to the inhibition of enzymatic activity associated with skin aging. The primary compounds possessing moisturizing properties are antioxidants (polyphenols and flavonoids), proteins, amino acids, and carbohydrates, which feature hydroxyl groups capable of forming hydrogen bonds with water (Ziemlewska et al., 2021). Zofia et al. (2020) showed that kombucha with coffee beans as a substrate could inhibit collagenase and elastase activities, improve skin hydration, and exert sunscreen effects. The concentrations of polyphenols, flavonoids, and caffeine in the fermentation broth considerably increased the inhibition of collagenase and elastase activities. The active ingredients in the coffee fermentation product, such as monosaccharides, amino acids, vitamins, polyphenols, and flavonoids, showed nourishing and soothing effects. The sunscreen properties of fermentates are mainly attributable to substances derived from flavonoids, polyphenols, anthocyanins, proteins, amino acids, and vitamins (Zofia et al., 2020).

### 3.9. Anti-cancer effects

**Figure 5:**
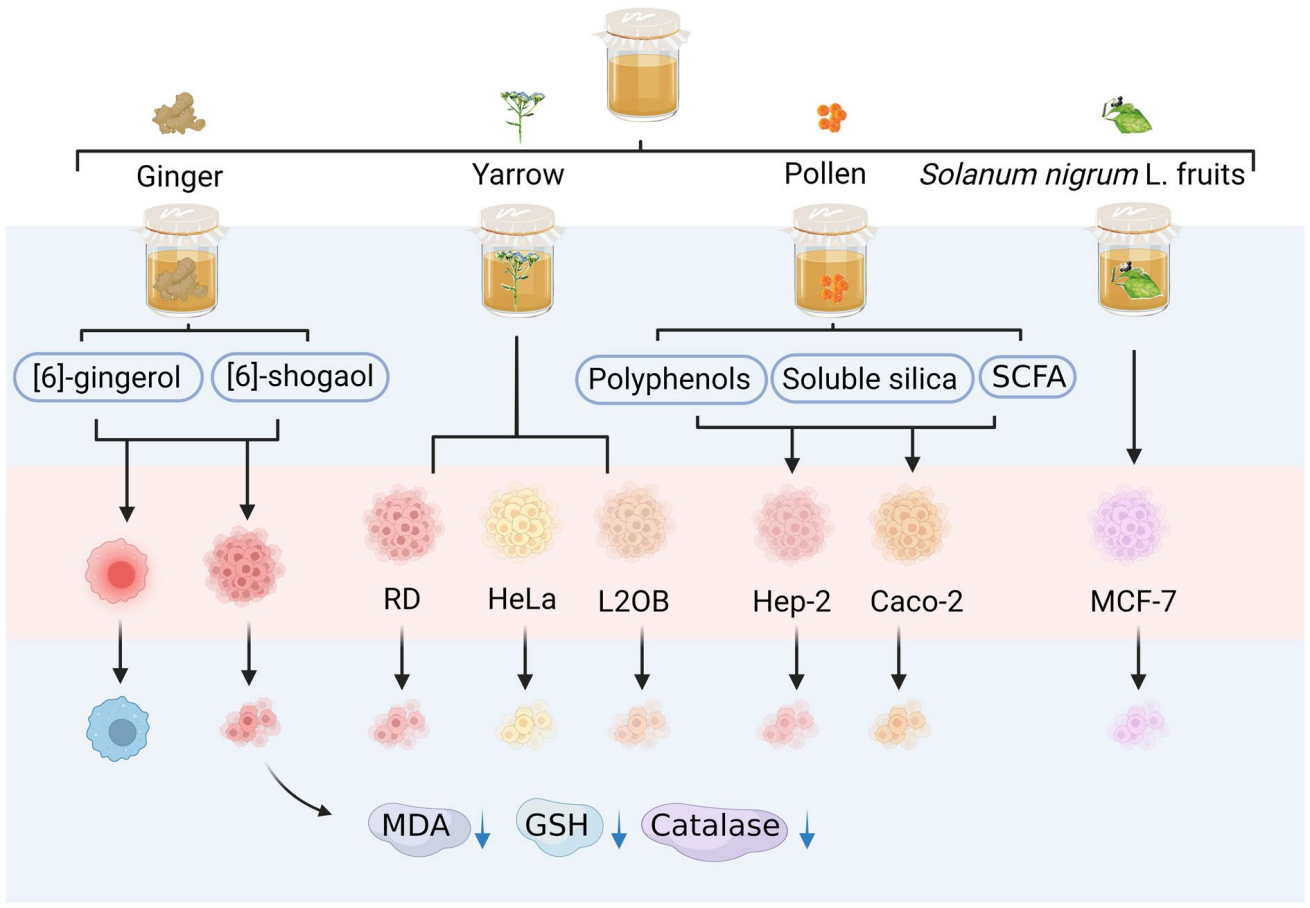
Anti-cancer activity of alternative substrates after fermentation. RD, rhabdomyosarcoma; HeLa, human cervical cancer Hep2c cells; L2OB, murine fibroblasts cancer cells; GSH, glutathione; MDA, malondialdehyde.

The consumption of kombucha can improve the immune system and enhance the ability of the body to fight cancer (Figure 5). Salafzoon et al. (2017) obtained samples from a 10-day kombucha fermentation of ginger infusion containing ginger bioactive components, such as gingerol and shogaol, which have anti-inflammatory and anti-tumour activities. These samples could inhibit tumour proliferation and stimulate apoptosis. Fermented ginger infusions reduced the activities of peroxidase, glutathione, and malondialdehyde in tumours, liver, and kidney homogenates (Salafzoon et al., 2017). Vitas et al. (2018) found that the fermentation broth of kombucha with yarrow as a substrate has antiproliferative activity against human rhabdomyosarcoma cells, human cervical cancer Hep2 cells (HeLa), murine fibroblasts (L2OB), and other tumour cells. Uțoiu et al. found that the fermentation broth of kombucha with bee pollen as a substrate exerted different levels of antitumour activity in Hep-2 and Caco-2 cells (Uțoiu et al., 2018). The fermented pollen exhibited elevated concentrations of bioactive compounds, including polyphenols, soluble silica substances, and short-chain fatty acids, thereby augmenting the health-promoting properties of pollen through kombucha fermentation (Uțoiu et al., 2018). Furthermore, the parameters for Kombucha fermentation were optimized to enhance the cytotoxic efficacy of the n-hexane fruit extract derived from *Solanum nigrum* L. against MCF-7 breast cancer cells (Ziska et al., 2019).

### 3.10. Other effects

The fermentation process of African mustard leaves by kombucha resulted in a notable enhancement in the overall phenolic and ethyl acetate levels, as well as the antioxidant capacity, when compared to the unfermented samples (Rahmani et al., 2019). In addition, kombucha fermentation increased anti-acetylcholinesterase activity; moreover, it reduced the cytotoxicity of *Brassica tournefortii* leaves and its inhibitory effect on xanthine oxidase (Rahmani et al., 2019). Zubaidah et al. conducted a study to examine the immunomodulatory properties of turmeric and black tea kombucha on BALB/c mice. The results of their investigation revealed that turmeric kombucha exhibited a significant enhancement in the adaptive immune response, mainly in the form of increased expression levels of CD4+, TNF-α, and IFN-γ, while enhancing the innate immune response, mainly in the form of decreased expression levels of CD68 and IL-6 (Zubaidah et al., 2021c). *L. barbarum* (Zhang et al., 2022), rose, and jujube kernels (Zhang et al., 2020) can contribute to cellulase activity when used as alternative fermentation substrates. With the use of winter savoury peppermint, stinging nettle, wild thyme, elderberry, quince (Vitas et al., 2020), and milk (Elkhtab et al., 2017), kombucha fermentation broth showed inhibitory activity against angiotensin-converting enzyme, and the resulting beverage showed antihypertensive potential.

**Figure 6:**
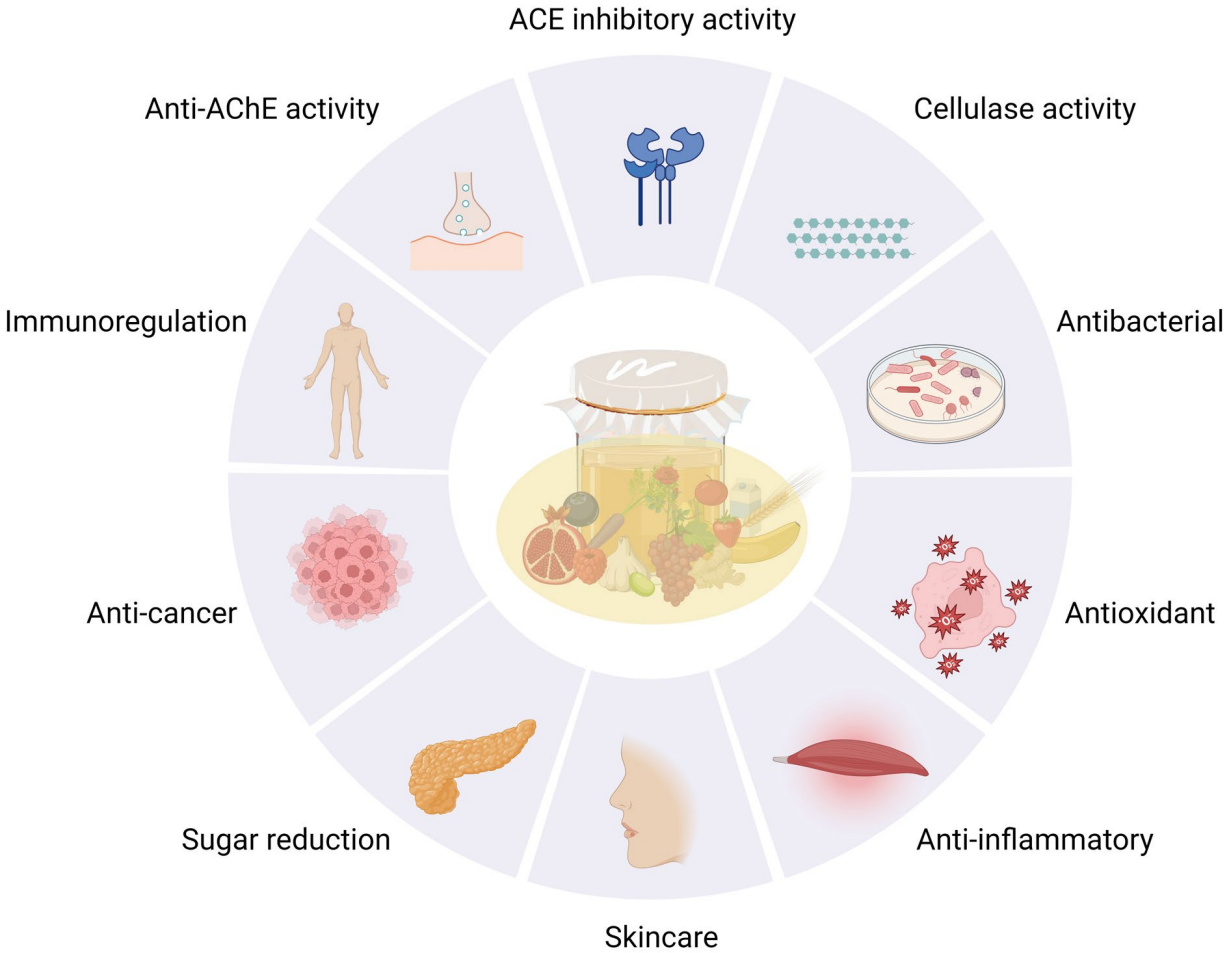
Pharmacological activity of different substrates in kombucha.

As shown in Figure 6, there have been numerous studies on the functions of kombucha. However, most have focused on functional efficacy, with minimal investigation into the underlying fermentation process.

### 3.11. Adverse effects

Instances of health issues resulting from the consumption of kombucha have been documented in scholarly literature, encompassing symptoms such as dizziness, headaches, allergic reactions, acidosis, and gastrointestinal ailments (Villarreal-Soto et al., 2018; Batista et al., 2022). These complications primarily arise from various factors, including excessive consumption, inadequate sanitation practices at home, contamination by pathogenic microorganisms, and susceptibility of immunocompromised individuals. For instance, pregnant women are advised against consuming kombucha due to its ethanol content, substantial acetic acid concentration, and potential presence of substances like heparin (Martínez-Leal et al., 2018; de Miranda et al., 2022). The excessive ingestion of kombuella in individuals with a history of excessive alcohol consumption has the potential to result in significant liver necrosis. Findings from pathological examination suggest a correlation between kombuella and liver damage in these patients, as evidenced by markedly elevated serum levels of aspartate aminotransferase and alanine aminotransferase, surpassing what would be expected solely from alcohol consumption (Sannapaneni et al., 2023).

## 4. Concluding remarks

We reviewed recent studies on the functional components of kombucha produced using alternative raw materials. The antibacterial and antioxidant effects of kombucha have been extensively studied. Kombucha contains diverse bioactive constituents, and the microorganisms responsible for the fermentation process exhibit intricate characteristics. At present, alternative raw materials for kombucha fermentation are mostly limited to foodstuffs, mainly for the development of beverages. This post-fermentation efficacy investigation identified the basic functions of kombucha, such as its antibacterial and antioxidant activities and focused on assessing the mixture of total acids, total phenols, and total flavonoids that could be beneficial.

Despite the numerous studies on kombucha, several unresolved issues remain. First, few studies have reported the inhibitory effects of fermentation broths produced from different substrates on drug-resistant, lethal, pathogenic, and typical inflammatory microbiota. Moreover, limited research has been conducted to investigate the constituent elements of distinct antibacterial and antioxidant compounds subsequent to the process of fermenting alternative raw materials. The refinement of its antimicrobial spectrum as well as the characterisation and purification of specific antimicrobial and antioxidant substances, are of great research significance.

Second, although kombucha has good anti-inflammatory properties, the experimental models used have been relatively homogeneous; clinical value assessments have not yet been carried out, and exploration of the anti-inflammatory mechanisms is still in the preliminary stages. Thus, more *in vivo* and *ex vivo* experiments are required to further elucidate the anti-inflammatory components, molecular mechanisms of action, and key functional strains to lay a solid theoretical foundation for subsequent clinical trials on the effects of kombucha on human health.

Third, insufficient scholarly investigation has been undertaken regarding the qualitative and quantitative analyses of particular substances, such as polyphenols and terpenoids, or the reaction processes of specific substances in mixed strains. The identification and purification of active ingredients after the fermentation of alternative raw materials, the metabolic pathways in kombucha, and the specific pharmacological mechanisms of action have not yet been extensively studied. It may be possible to broaden the range of raw materials for kombucha fermentation to traditional Chinese medicines or pure monomeric compounds, similar to the development of specific pharmaceutical fermenters. The metabolic reaction pathways and products of specific substances during strain fermentation could be supplemented. Alternatively, controlling the process to increase the yield of the main drug could retain the advantages of kombucha, such as containing many beneficial ingredients and natural antibacterial properties, which have promising applications in cosmetics and pharmaceuticals.

Future research should focus on the predominant fermentation strains and their interactions to develop improved and consistent artificial agents that can aid in standardising kombucha production.

## Author contributions

JS: Conceptualization, Data curation, Formal analysis, Funding acquisition, Investigation, Methodology, Project administration, Resources, Supervision, Validation, Visualization, Writing – original draft, Writing – review & editing. QTan: Data curation, Formal analysis, Investigation, Methodology, Software, Validation, Visualization, Writing – original draft. QTang: Data curation, Formal analysis, Investigation, Methodology, Software, Writing – original draft. ZT: Data curation, Formal analysis, Investigation, Methodology, Software, Writing – original draft. MY: Conceptualization, Data curation, Formal analysis, Funding acquisition, Investigation, Project administration, Supervision, Validation, Visualization, Writing – review & editing.

## Funding

The author(s) declare financial support was received for the research, authorship, and/or publication of this article. This work was supported by the National Natural Science Foundation of China (81901243), the Fujian Provincial Regional Development Project (2021N3005), and the Natural Science Foundation of Fujian Province, China (2021J01204).

## Conflict of interest

The authors declare that the research was conducted in the absence of any commercial or financial relationships that could be construed as a potential conflict of interest.

## Publisher’s note

All claims expressed in this article are solely those of the authors and do not necessarily represent those of their affiliated organizations, or those of the publisher, the editors and the reviewers. Any product that may be evaluated in this article, or claim that may be made by its manufacturer, is not guaranteed or endorsed by the publisher.
